# Multichannel acoustic source and image dataset for the cocktail party effect in hearing aid and implant users

**DOI:** 10.1038/s41597-020-00777-8

**Published:** 2020-12-17

**Authors:** Tim Fischer, Marco Caversaccio, Wilhelm Wimmer

**Affiliations:** 1 Department of ENT, Head and Neck Surgery, Inselspital, Bern University Hospital, University of Bern, Bern, 3008 Switzerland; 2 Hearing Research Laboratory, ARTORG Center for Biomedical Engineering Research, University of Bern, Bern, 3008 Switzerland

**Keywords:** Machine learning, Computer science, Biomedical engineering, Auditory system

## Abstract

The Cocktail Party Effect refers to the ability of the human sense of hearing to extract a specific target sound source from a mixture of background noises in complex acoustic scenarios. The ease with which normal hearing people perform this challenging task is in stark contrast to the difficulties that hearing-impaired subjects face in these situations. To help patients with hearing aids and implants, scientists are trying to imitate this ability of human hearing, with modest success so far. To support the scientific community in its efforts, we provide the Bern Cocktail Party (BCP) dataset consisting of 55938 Cocktail Party scenarios recorded from 20 people and a head and torso simulator wearing cochlear implant audio processors. The data were collected in an acoustic chamber with 16 synchronized microphones placed at purposeful positions on the participants’ heads. In addition to the multi-channel audio source and image recordings, the spatial coordinates of the microphone positions were digitized for each participant. Python scripts were provided to facilitate data processing.

## Abstract (summary)

**Table:** 

Measurement(s)	acoustic Cocktail Party scenarios • acoustic composition of speech • Noise
Technology Type(s)	head-mounted microphones
Factor Type(s)	head shape of the participants • acoustic Cocktail Party scenarios played
Sample Characteristic - Organism	Homo sapiens

Machine-accessible metadata file describing the reported data: 10.6084/m9.figshare.13181921

## Background & Summary

When was the last time you were at a party or a crowded restaurant? Surely you remember the babble of voices and the presence of background music at such events. But when you hear your own name in one of the conversations or spot someone you know, your perception suddenly changes: your brain filters out the voices you are interested in with amazing effectiveness^1^. This is only possible because irrelevant voices and background noises are suppressed at the same time^2^. The underlying process is called selective hearing and becomes more difficult when the party becomes larger and the number of competing sound sources increases^3,4^. Another depictive term describing the corresponding example is the Cocktail Party Effect or Problem^5^. The speech understanding in such complex acoustic situations differs significantly between people with normal hearing and people wearing cochlear implants (CIs) or hearing aids. This is why the acoustic Cocktail Party scenario is an open field of research relevant for both audiology and signal processing sciences^1,6,7^. Due to the growing popularity of voice assistants (e.g. Amazon Echo or Google Home) the majority of available Cocktail Party scenario datasets focus on recordings from distant microphone arrays^8–17^. Compared to the rapidly increasing number of hearing aid and implant users worldwide^18^, acoustic Cocktail Party datasets with microphones located at the human head are scarce and limited with respect to the number of human participants^19–23^, the number or placement of microphones^9,10,20–25^ or the acoustic stimuli that do not cover varying signal-to-noise ratios (SNRs) or Cocktail Party scenarios^19,24,26,27^. In addition, many available datasets lack specifications of the microphones’ positions and the spatial arrangement of the individual sound sources, which makes it difficult to characterize the occurring interaural or inter-microphone time and level differences^28,29^. Time and level difference information are particularly relevant for sound source localization or separation techniques^30,31^.

Recent advancements in deep neural networks have led to a substantial improvement in the performance of automatic speech recognition (ASR) and blind source separation (BSS) tasks^32–34^. Although unsupervised learning approaches exist^35,36^, many of the currently applied deep learning algorithms are trained by minimizing the distance between the estimated speech or target signal and the clean reference signal of the dataset. Often the evaluation of word error rates (WERs) are desired, which requires text annotations of the speech files in addition to the reference audio speech signals^9,10^.

Users of hearing aids or CIs have not been the explicit focus of the datasets covering acoustic Cocktail Parties so far. The dataset presented in this data descriptor aims to fill this gap and contains 6400 multi-channel recordings (total duration of 08 h 48 min) of 160 acoustic Cocktail Party scenarios measured with microphones located on the head of 20 different individuals. Each of the 160 Cocktail Party scenarios refers to an unique spatial arrangement of speech and noise sources with various intensity levels. In addition, 49538 recordings (with a total duration of 80 h 25 min) obtained from microphones placed on a head and torso simulator with 15224 different Cocktail Party scenario arrangements were recorded, including separate recordings of the corresponding noise and speech images. A brief overview of data descriptors related to the one presented here can be found in Table 1. The main goal of this dataset is to provide comprehensive data to the scientific community to facilitate the development of techniques to improve speech understanding of hearing aid users in complex acoustic scenarios. The structure and content of the dataset allows detailed evaluations of the performance of audio signal processing algorithms. In combination with the supplied code and open source spatialization toolkits^37^, effects such as varying reverberation may be added. In general, it is easier to introduce reverberation than to de-reverb recordings. In addition to the multi-channel source and image audio files, our dataset includes metadata to achieve transparency and easy usability of the dataset. The possible applications of the dataset include, but are not limited to: multi-channel audio (blind) source separation and localization techniques, automatic speech recognition or speech enhancement especially for the challenging multiple concurrent speakers case^38^, algorithmic or human word recognition performance evaluation, audiological assessments such as spatial release from masking^2,39^, creation of virtual acoustic scenes and (unsupervised) domain adaption^11^.

**Table 1:** A comparison of the presented data descriptor with existing literature.

Dataset	Microphone setup	Participants	Stimuli	Recording time	Metadata
Bern Cocktail Party (BCP)	16 microphones distributed on the head, the ears and 2 cochlear implant audio processors including transmissions coils.	20 adults (13 male, 7 female) and a dummy head.	Clean speech taken from the LibriTTS corpus^40^, overlays of speech, music and babble noise recorded in an acoustic chamber (English). Playback with up to 12 loudspeakers.	89 hours	Microphone distance matrices and absolute coordinates, head and pinna measurements, overall and channel specific SNR values, transcriptions for all speech sources (including babble noises), audio source files, python scripts for data processing, microphone array PCB layouts and schematics, 2 to 100 speaker babble noise files with transcribed speech
DiPCo - Dinner Party Corpus^8^	39 microphones (4 close-talk microphones and 5 × 7 far-field microphones distributed in the recording room).	32 adults (19 male, 13 female), 4 per session	Natural conversation around a dining table with music playback at given time marks (English).	10 sessions with durations from 15 to 45 minutes	Human labeled transcripts, geometric arrangement of the 7-microphone array, recording room layout
The fifth CHiME Challenge Dataset^9^	6 microphone arrays (min. 2 in each room) and 4 binaural microphone pairs.	4 per session	Natural conversations recorded in 20 homes (English).	20 sessions with a minimum duration of 2 hours each	Human labeled transcripts
Libri-Adapt: Dataset for Unsupervised Domain Adaptation^11^	6 different recording devices with 1 to 7 microphones. Devices were placed 15 cm from the loudspeaker.	No human or dummy head recordings	English speech (3 accents) in the presence of 4 noise types, all taken form the LibriSpeech^61^ corpus. Playback from 1 loudspeaker.	7200 hours (6 microphones x 3 accents x 4 environments x 100 hours)	Technical specification of the microphones used
The Sweet-Home speech and multimodal corpus for home automation interaction^13^	7 microphones in a smart home with 4 rooms.	4 dataset subsets with 11 to 23 participants. 1 participant per session	French speech (with partly added noise), noise of living activities, vocal orders for home automation.	26 hours	SNR values, location of the participants and their activity, transcribed speech, transcribed home automation traces
VoiceHome-2, an extended corpus for multichannel speech processing in real homes^14^	8 microphones positioned on a cube which was placed in defined room positions.	12 adults, 3 per session	Clean and spontaneaous speech recorded in 12 rooms in 4 different homes, Noise-only segments (French).	5 hours	Microphone and speaker positions, transcribed speech, room impulse responses, noise and room types
The CHIL audiovisual corpus for lecture and meeting analysis inside smart rooms^15^	64-channel linear microphone array, 3 × 4-channel T-shaped microphone clusters and 3 table-top microphones placed in a smart room. In addition, all speakers wore close-talking microphones.	3 or more per session	Natural converstaions during lectures or meetings recorded in 5 smart rooms (English with accents).	86 lectures with a duration of approximately 30 minutes each	Manual annotations of audio and visual modalities
Voices Obscured in Complex Environmental Settings (VOICES) corpus^16^	12 distant microphones distributed in a room.	No human or dummy head recordings	English speech taken from the LibriSpeech corpus^61^ in 2 furnished rooms with background noise. Playback from 4 loudspeakers.	120 hours	SNR values, microphone foreground loudspeaker(s) distance, microphone locations
The DIRHA-English corpus^17^	1 studio microphone and 62 microphones distributed in 5 rooms of a flat.	24 adults (12 male, 12 female), 1 per session	English sentences that were played or read aloud in the living room of the apartment.	11 hours	Clean speech recordings, transcribed speech
Acoustic Impulse Responses for Wearable Audio Devices^19^	80 microphones spread across a human body and 80 microphones affixed to wearable accessories.	1 human subject, 1 mannequin	Frequency sweeps in an acoustically treated recording space with 1 loudspeaker.	n.a.	Acoustic impulse responses
Database and Target Response Correction Functions for Various Device Styles^27^	9 microphone locations on 5 different hearing device styles.	16 subjects (10 male, 6 female) and 2 dummy heads	Frequency sweeps in an anechoic chamber with 91 loudspeakers.	n.a.	Head Related Transfer Functions (HRTFS) and Target Response Correction Functions (TRCFs)
EU-project: HearCom^23^	6 microphones distributed on 2 hearing aid dummies.	Dummy head	Noise and speech in various rooms and outdoor environments (English).	n.a.	Extensive performance measures, azimuth of sound sources, mixtures of noise and speech, spatial information from the multi-microphone recordings

## Methods

### Participants and test procedure

**Fig. 1:**
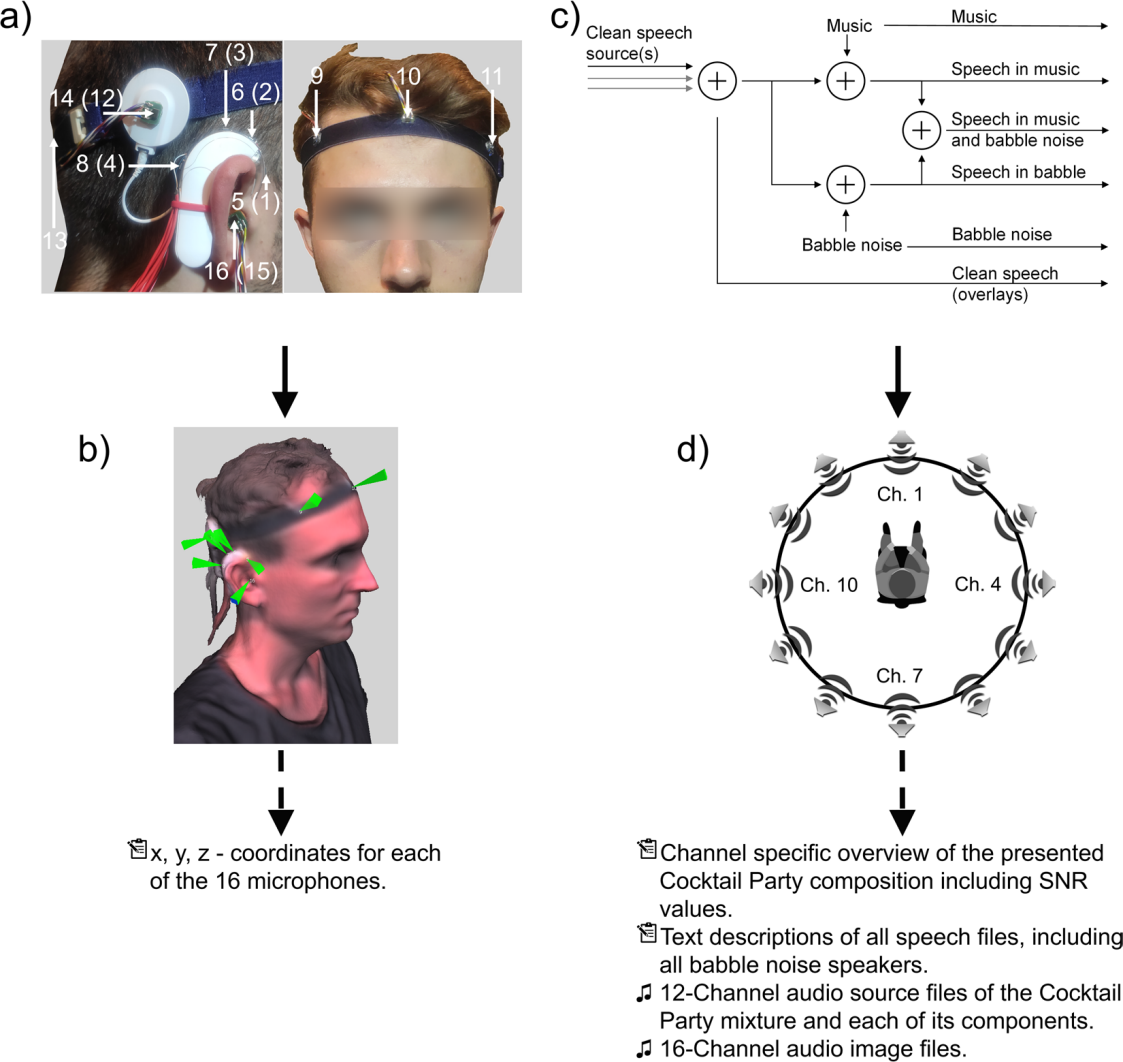
Schematic overview of the data acquisition process. (**a)** On-head locations of the 16 microphones. The numbers refer to the assignment of the microphone channels in the multi-channel recording audio files (shown for the right ear). Numbers in brackets refer to the contralateral assignment of the microphone channels. A text-based description of the numerical marker labels can be found in Table 2. (**b)** Example of an anonymized three-dimensional full head scan. The markers depict the microphone positions from which the X,Y and Z coordinates were extracted. A text-based description of the spatial coordinates can be found in Table 2. (**c)** Flowchart to illustrate the structure of the generated 12-channel audio mixtures. The output signals of this flowchart show the generated source and recorded .wav files. Not illustrated are the created and recorded sub-combinations of the speech and noise signals (e.g. music and babble) that make up the mixture. (**d)** Arrangement of the 12 loudspeakers positioned in a circle around the seated participant. Each acoustic Cocktail Party scenario was generated by a 12-channel overlay of speech and noise signals and played back through the illustrated speaker configuration. The persons depicted have explicitly agreed to be included in this figure.

The data was collected from 7 female and 13 male adults with a mean age of 30.6 ± 5.4 years. The participants’ task was to sit in the center of a horizontal circular loudspeaker setup (Control 1 Pro, JBL, Northridge, USA) while pre-defined acoustic Cocktail Party scenarios were presented and simultaneously recorded by 16 microphones (ICS-40619, TDK, Tokyo, Japan) (see Fig. 1a and Table 2). After the microphones were attached to the participants’ head, the relative positions of the microphones were captured with a 3D scanner (Structure Core, Occipital Inc., USA).

**Fig. 2:**
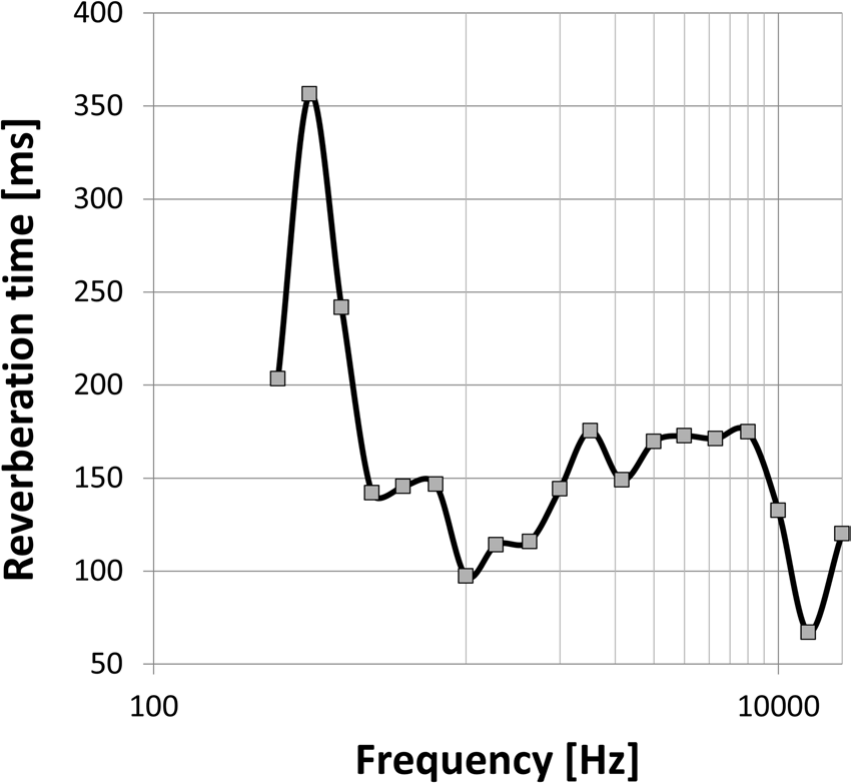
Frequency dependent reverberation times in milliseconds of the acoustic chamber used for this dataset.

Each of the 20 recording sessions of the varying acoustic Cocktail Party scenarios contained audio files of 26 minutes. Before the recording, the participants were instructed to sit as still as possible during the measurement. In addition to the data from real human participants, a head and torso simulator (Brüel & Kjær, Type 4128, Nærum, Denmark) was used to capture 80 hours of acoustic Cocktail Party scenarios.

The study was designed in accordance with the Declaration of Helsinki, reviewed by the cantonal Ethics Committee Bern (Switzerland) and declared not subject to approval. Written informed consent was obtained from all participants.

### Audio source files

The Cocktail Party scenarios are composed of acoustic overlays from English speech, music, and babble noise^40,41^. Each mixture consists of a 12-channel .wav file with a sample rate of 44.1 kHz and an audio bit depth of 24 bit.

For the composition of the multichannel Cocktail Party files, each audio file was set to a specific level in loudness units realtive to full scale (LUFS). The measure of LUFS is recommended by the European Broadcasting Unit (EBU) and is intended to reflect the perceptual estimation of loudness. Compared to classical SNRs derived from levels measured in a-weighted decibel (dBA) or dB sound pressure level (SPL), the calculation with LUFS better correlates with human perception, because it is silence-invariant, and little sensitive to downsampling. The loudness measurement algorithm was defined as specified in the ITU-R BS.1770-4 guidelines^42^. Our choices of the LUFS allow the dataset to cover a wide range of noise scenarios with SNRs values ranging from clean speech to −18.1 dB with multiple overlapping speakers in babble noise and music (see Fig. 1c and Tables 3 to 8). The SNR refers to the ratio of the level of one speaker with respect to the level of the remaining audio components of the mixture. In accordance with the ITU-R BS.1770 guidelines^42^, all channels were regarded as being incoherent and no channel weighting was applied.

For audio mixing, the files were selected such that no clipping occurred. In addition, a fade-in and fade-out of 100 ms was applied to all created audio files.

Since the aim of this dataset is to provide recordings of Cocktail Party scenarios which are traceable in their composition (see Table 9), each multi-channel audio source compilation is accompanied by the following additional data:

- Multi- and single channel audio source files of which the Cocktail Party is composed of.
- Channel specific text descriptions of the dominant speaker(s).
- Channel specific text descriptions of every speaker in the babble noise.
- Tabular overview of the audio channel configuration and applied level modifications for each channel.

The items in the above list are described in more detail in the following sections.

#### Speech sources

All speech files used in this dataset were selected from the “clean” LibriTTS^40^ corpus and are characterized by an SNR^43^ of at least 20 dB. If a Cocktail Party scenario contains multiple speech sources, speech files with a maximum offset in duration of 250 ms were combined. For all Cocktail Party recordings, the duration of the mixtures were defined by the duration of the speech files. Since all speech files are provided with a text description^40^, the dataset is suitable for single and multi-speaker ASR tasks in varying spatial arrangements and noise settings. Although the LibriTTS corpus is not part of the established clinical speech intelligibility test sets, speech intelligibility tests can also be performed with this data descriptor. To ensure suitability for human perceptual measurements, only files with a duration between 3 s and 10 s were included, resulting in a pool of 22063 different speech files.

#### Background music

Background music contains a random selection from the Musan “popular” corpus^41^. The “popular” section was chosen, as this type of music is most common in everyday acoustic Cocktail Party scenarios. To avoid intro and outro sections, the first and last 30 s were excluded from the music files. The remaining parts were sliced in excerpts of 10 s each to ensure a sufficient length with respect to the longest possible speech file duration. In total, a pool of 3229 different music snippets was included.

#### Babble noise

Babble noise is considered as one of the most suitable noises for masking speech^44^. It is defined as an overlay of at least 2 simultaneous speech sources. In our dataset this overlay was created by multi-channel combinations of 2-person babble noise, resulting in multi-speaker babble noise. To be compatible with the longest possible speech file durations, the first 10 s of speech files from the “clean” LibriTTS^40^ corpus were combined to a babble noise. For every created babble noise file, a corresponding text file exists which allows the assignment to the corresponding speech files of which the babble noise is composed of. In total, a pool of 3538 two-speaker babble files was created. In addition to the two-speaker babble files used for the creation of the Cocktail Party scenario files, a total of 6049 babble noises with 4 to 100 overlaying speakers were created and are part of this dataset.

### Measurement setup

Each 12-channel audio file was presented and recorded inside a sound-attenuated acoustic chamber (6.4 × 4.3 × 2.2 m^3^) with an approximate average reverberation time of 160 ms for frequencies between 0.25 and 16 kHz (see Fig. 2). Twelve loudspeakers (Control 1 Pro, JBL, Northridge, USA) were equidistantly arranged in a circle with a radius of 1.1 m around the center point, i.e. the participant’s head or a head and torso simulator (Brüel & Kjær, Type 4128, Nærum, Denmark) at ear level (see Fig. 1d)^45,46^. For our measurement setup and the presented stimuli, the critical distance is approximately 1.9 m. The directivity index was chosen with a value of *Q* = 3 corresponding to the spectral power density of human speech or babble noise^47^ and the directivity index diagram shown in the Control 1 Pro (JBL) loudspeaker data sheet. The loudspeaker data sheet also contains a diagram that describes the frequency response.

One channel of the 12-channel dataset file was assigned per loudspeaker using a multi-channel audio interface (MOTU 16 A, Motu, Cambridge, USA). All stimuli were played back and simultaneously recorded with a Python (Ver. 3.7) script using the sounddevice library (https://python-sounddevice.readthedocs.io/en/0.3.15/). Before recording, all loudspeakers were calibrated to 65 dB SPL (normal conversation level), with a 100 overlaid speaker babble file played at −30 LUFS. The level of −30 LUFS corresponds to the baseline level for the speech files used in this dataset.

### Microphones setup

To capture the (enhanced) auditory space of the presented Cocktail Party scenarios for normal hearing participants as well as hearing aid and CI users, 16 omnidirectional top-port micro-electro-mechanical systems (MEMS) microphones (ICS-40619, TDK, Tokyo, Japan) were integrated into custom circuit boards and placed at purposeful positions on the participants’ heads (see Fig. 1a). MEMS microphones were selected for their small form factor (3.5 × 2.7 × 0.9mm), flat frequency response up to 20 kHz and low noise floor of −105 dB V. The frequency response of the ICS-40619 top-port MEMS microphone can be found in the respective data sheet.

The arrangement of the microphones consists of 2 × 4 microphones, attached on a CI audio processor dummy (Sonnet, Med-El GmbH, Innsbruck, Austria) and 2 × 1 microphones attached onto the corresponding transmission coils (see Fig. 1a)). Originally this CI audio processor incorporates 2 of the 4 microphones which we placed at the processor (channel IDs 2, 3 (left side) or 6, 7 (right side) as described in Table 2). In addition to the 10 microphones placed on the CI audio processors, two microphones were placed at the entry of each ear canal to capture the frequency transformations of the incoming sound waves caused by the pinna^29,48–51^. The remaining 4 microphones were attached to an elastic headband as used in Gawliczek *et al*.^52^. After the headband was put on, the microphones were located at the front, the back, the right temple and the left temple positions.

**Table 2:** The table shows the assignment of the 16 microphone positions to the respective channel ID.

Channel ID	Description of the microphone position
1	Left audio processor. Facing forward.
2	Left audio processor. Facing top/forward.
3	Left audio processor. Facing top/back.
4	Left audio processor. Facing back.
5	Right audio processor. Facing forward.
6	Right audio processor. Facing top/forward.
7	Right audio processor. Facing top/back.
8	Right audio processor. Facing back.
9	Right temple.
10	Front.
11	Left temple.
12	Left transmission coil.
13	Back.
14	Right transmission coil.
15	Left Ear. Entry of the ear canal.
16	Right Ear. Entry of the ear canal.

The recorded microphone signals were preamplified (Behringer ADA8200, Music Tribe Holding, Makati City, Philippines) and transmitted to a personal computer via an audio interface (MOTU 16 A, Motu, Cambridge, USA). The resulting .wav files were synchronously sampled at 44.1 kHz with an audio bit depth of 32 bit.

#### Acquisition of the spatial coordinates of the microphones

To increase the traceability of the 16-channel microphone recordings, the 3D positions of the microphones were recorded for each participant and the head and torso simulator. In addition to the interaural or inter-microphone time differences between the microphones, the so-called head shadow effect between the microphones can be related to the anatomy of the test person’s head^29^. The head shadow effect creates interaural level differences and plays a significant role in binaural hearing^53^. Head-related anatomical measurements were performed using the 3D head scans and include the head circumference and width. Furthermore, the ear width and length was measured. The ear width was defined as the distance from tragus to helix and the ear length as the distance between the highest point of the auricle and the lowest point of the ear lobe.

The 3D model was obtained by a full head scan (Structure Core, Occipital Inc., USA) and further analyzed using the open-source software Meshlab (ISTI-CNR, Rome, Italy). Using the provided spatial coordinates, the distances between the microphones can be defined. The authors consider an annotation uncertainty with a standard deviation of ±1.5 mm due to color and structural unevenness of the acquired 3D models to be reasonable.

An illustration of a 3D model can be found in Fig. 1b.

#### Audio source multi-channel files

All audio source multi-channel files were mixed with the Python audiosegment module (https://audiosegment.readthedocs.io/en/latest/audiosegment.html) which exposes a wrapper of a pydub.AudioSegment object (https://github.com/jiaaro/pydub/). In the specific case of a channel overlay of 2 or more sound sources, such as with the provided multi-speaker babble files, the function *overlay* from the pydub.AudioSegment library was used. The LUFS level calibration of the channels in accordance to the ITU-R BS.1770-4 guidelines^42^ was achieved with the use of the Python module pyloudnorm (https://github.com/csteinmetz1/pyloudnorm). For the playback and simultaneous recording of the provided multichannel source files the Python function *playrec* of the sounddevice library was used.

#### Dataset processing

We provide a Python function to extract a user-specific list of audio files from the database. This allows, for example, to extract only those recordings from all files in which 2 speakers and background music are present. An explanation of the search parameters can be found in the file config_template.py. An example file extraction query is provided in the file example_get_desired_wav_list.py. In order to extract only files of the participant ID_01 for example, the variable FOLDER_PREFIX must be set to the corresponding folder name of ’Human_Subjects_Audio_ID_01’.

If a specific direction of origin of speech material is desired, the script example_get_von_mises.py can be used to extract the corresponding files from the dataset. The function parameters *σ* and *μ* allow the parameterization of a circular normal distribution. In the provided example, with *σ* = 55 and *μ* = 0, a dataset can be created in which the target speech sources occur mainly in the frontal direction.

To ensure the usability of the source and recording files also for single-channel applications, a very common scenario in speech enhancement tasks^54^, the multi-channel files can be transformed to mono files with acoustically overlaid channels. The transformation of multi-channel files to mono audio files is shown and performed in the provided Python file example_transform_to_mono.py.

Since we wanted to mitigate the influence of microphone preamplifier noise in our recordings, some users might consider the original recordings as too quiet. Therefore, we provided a script in the file example_adjust_loudness.py to adjust the level of a user-defined list of wav files.

#### Hardware development

The schematics, Gerber files and PCB layouts of the microphone arrays developed for this study were designed with EAGLE 9.5 (Autodesk Inc., San Rafael, USA) and are attached to the dataset. To view the files, the free version of EAGLE can be downloaded from the homepage of Autodesk Inc. The provided files allow to have the used microphone hardware replicated by a PCB Assembly Service.

## Data Records

All data created in this research project are accessible from the figshare repository^55^. The dataset contains a collection of acoustic cocktail party configurations (CPCs) with metadata and babble noise files with 2 to 100 parallel speakers. In addition to the audio files, 3D head scans were acquired for each participant and the head and torso simulator. The spatial coordinates of the microphone positions are provided in a.csv file. Furthermore, the Euclidean distances between the microphones were calculated for each participant and averaged over all participants; they are also available as.csv files. All position and distance data of the microphone coordinates are given in millimeters. In addition to the microphone distance matrices and absolute coordinates, the tilt of the microphone headband (see Fig. 1a) in relation to the “Frankfurt Plane” was calculated for all participants and added as a .csv file.

### Real human head recordings

The configurations of active speech sources that were used in the Cocktail Party dataset involving recordings with human participants are shown in Table 3. In total, N_s_ = 16 configurations of speech sources were created for the dataset recorded with human participants. Table 4 summarizes the different background noise configurations that were overlaid to the speech source configurations. In total, N_N_ = 10 noise configurations were combined with each of the N_S_ configurations. For the human participants dataset, this results in a total of N_CPC_ = N_S_·N_N_ = 160 different CPCs. For each of the 20 participants, 2 samples of CPCs were recorded. Each sample of the CPCs is unique in its acoustic composition of speech and noise components. For all tested participants, N = 6400 Cocktail Party mixture recordings with an overall duration of 08 h 48 min and an average duration per sample of 5.0 ± 0.6 seconds are available. The speech files of the CPCs consist of 5955 different sentences spoken by 305 different speakers. Each speech file is used 2.2 times with a standard deviation of 1.2. The percentage of speech files spoken by a female speaker is 55%. The resulting speaking time of 55% of female speakers demonstrates a balanced gender ratio. The noise components of the mixtures consist of 3538 different babble noises and 3229 music files. Each babble noise was used 10.9 ± 0.4 times and each music file was used 2.8 ± 1.9 times. An overview of the SNR of the source files is given in Table 5. The azimuth dependent channel weighted SNR as specified in ITU-R BS.2051^56^ are provided in the .csv files which are described in Table 10.

**Table 3:** Spatial measurement configurations for active speech sources in the human measurements.

Azimuths of the speech sources (°)	Level (LUFS)
−90	−30
0	−30
90	−30
150	−30
210	−30
0,90	−30
0,−90	−30
0,30	−30
−30,0	−30
−30,30	−30
0,180	−30
−30,0,30	−30
−30,90,210	−30
0,120,240	−30
30,150,270	−30
−90,0,90	−30

The first column describes the azimuth of the speech sources. LUFS values refer to a channel gain of 1.0 (no gain)^42^.

**Table 4:** Spatial measurement configuration for active noise sources in the human measurements.

Azimuth music (°)	Azimuth babble (°)	Level (LUFS) Music/Babble	Noise Filename prefix
No Noise	No Noise	No Noise	CS1
90		−45/n.a.	MU2
180		−45/n.a.	MU3
270		−45/n.a.	MU4
0,90,180,270		−45/n.a.	MU7
	0, 30, 60, …, 330	n.a./−55	BA5
90	0, 30, 60, …, 330	−45/−55	MB2
180	0, 30, 60, …, 330	−45/−55	MB3
270	0, 30, 60, …, 330	−45/−55	MB4
0, 90, 180, 270	0, 30, 60, …, 330	−45/−55	MB7

The first and the second column describe the azimuth of the background music sources and the babble noise sources. LUFS values refer to a channel gain of 1.0 (no gain)^42^ and are applied to one source each. The fourth column indicates the filename prefix for this specific noise setting.

**Table 5:** The data shows an overview of the SNR values and one-standard deviations for the multi-channel source files in the human measurements.

Noise	SNR (Loudness setting ID = 0)		
Filename prefix	1 speaker	2 speakers	3 speakers
CS1	no noise	0.04 ± 0.1	−2.7 ± 0.2
MU2	15.1 ± 0.1	−0.01 ± 0.01	−2.8 ± 0.2
MU3	15.1 ± 0.1	0.01 ± 0.1	−2.8 ± 0.2
MU4	15.1 ± 0.1	−0.01 ± 0.1	−2.8 ± 0.2
MU7	9.1 ± 0.1	−0.2 ± 0.2	−2.9 ± 0.2
BA5	14.5 ± 0.1	−0.1 ± 0.1	−2.8 ± 0.2
MB2	11.8 ± 0.1	−0.1 ± 0.2	−2.9 ± 0.2
MB3	11.8 ± 0.1	−0.1 ± 0.2	−2.9 ± 0.2
MB4	11.8 ± 0.1	−0.1 ± 0.2	−2.9 ± 0.2
MB7	8.1 ± 0.1	−0.3 ± 0.3	−3.0 ± 0.2

The noise-filename prefixes are defined in Table 4. All speech sources were set to the same level (Loudness setting ID = 0, −30 LUFS), as defined in Tables 3 or 9.

An overview of the demography as well as the head circumferences, head widths, pinna lengths and pinna widths of all participants can be found in the “Demography.csv”.

### Head and torso simulator

The data in Table 6 shows the different configurations of active speech sources that were included in the created CPCs. In total, N_S_ = 130 configurations of speech sources were recorded using the head and torso simulator. Table 7 shows the different background noise configurations that were overlaid with the speech source configurations. In total, N_S_ = 20 noise configurations were combined with each of the N_S_ configurations. For the head and torso simulator, this results in N_CPC_ = N_S_· N_N_ = 2600 different CPCs. Each CPC was recorded with at least 3 different combinations of speech and noise files. Only if the overall duration t_samples_ was smaller than 15 s, more than 3 samples were recorded, until t_samples_>15 s were measured. In total, N = 8449 CPC recordings with an overall duration of 13 h 44 min and an average duration per sample of 5.9 ± 2.0 seconds were recorded. Every CPC recording is unique in its acoustic composition of speech and noise components. Since CPCs with multiple speech sources include multiple target signals, a total of 21086 unique target signals can be evaluated using our Head and Torso Simulator data. For CPCs with more than 1 speaker, all speech with corresponding noise source combinations were additionally recorded, resulting in an overall sum of 49538 different recordings with at least 1 speech source present.

**Table 6:** Spatial measurement configurations for active speech sources in the head and torso simulator measurements.

Azimuths of the speech sources (°)	Number of shifts	Level (LUFS)
0	12	−30
0, 30	12	−30, −30
0, 90	12	−30, −30
0, ± 180	6	−30, −30
0, ± 180	12	−15, −30
0, 30, 60	12	−30, −30, −30
0, 90, −90	12	−30, −30, −30
0, 120, −120	4	−30, −30, −30
0, 90, −90	12	−15, −30, −15
0, 90, −90	12	−30, −15, −30
0, 120, −120	12	−15, −30, −15
0, 120, −120	12	−30, −15, −30
Total number of speech settings (*N*_*S*_)	130	

The first column describes the azimuth of the speech sources. A shift refers to the displacement of the speech sources with a step size of 30° in clockwise direction. Shifts were performed after the data for the setting for the measurement configuration has been recorded. LUFS value refer to a channel gain of 1.0 (no gain)^42^.

**Table 7:** Spatial measurement configuration for active noise sources in the head and torso simulator measurements.

Azimuth music (°)	Azimuth babble (°)	Level (LUFS) Music/Babble	Noise Filename prefix
No Noise	No Noise	No Noise	CS1
0		−45/n.a.	MU1
90		−45/n.a.	MU2
180		−45/n.a.	MU3
270		−45/n.a.	MU4
0, 180		−45/n.a.	MU5
0, 270		−45/n.a.	MU6
0, 90, 180, 270		−45/n.a.	MU7
	330, 0, 30	−45/−55	BA1
	60, 90, 120	−45/−55	BA2
	150, 180, 210	−45/−55	BA3
	240, 270, 300	−45/−55	BA4
	0, 30, 60, …, 330	n.a./−55.	BA5
0	0, 30, 60, …, 330	−45/−55	MB1
90	0, 30, 60, …, 330	−45/−55	MB2
180	0, 30, 60, …, 330	−45/−55	MB3
270	0, 30, 60, …, 330	−45/−55	MB4
0, 180	0, 30, 60, …, 330	−45/−55	MB5
0, 270	0, 30, 60, …, 330	−45/−55	MB6
0, 90, 180, 270	0, 30, 60, …, 330	−45/−55	MB7

The first and the second column describe the azimuth of the background music sources and the babble noise sources. LUFS values refer to a channel gain of 1.0 (no gain)^42^ and are applied to one source each. The fourth column indicates the filename prefix for this specific noise setting.

The speech files of the CPCs consist of 19390 different sentences spoken by 317 different speakers. Each speech file is used 1.1 times with a standard deviation of 0.3. The percentage of speech files spoken by a female speaker is 54.6%. The resulting speaking time of 54.1% of female speakers demonstrates a balanced gender ratio.

The noise components of the mixtures consist of 3538 different babble noises and 3229 music files. Each Babble noise was used 12.8 ± 0.6 times and each music file was used 3.1 ± 1.8 times.

In addition to the recordings of the CPCs, each noise and speech component of the mixture as well as combinations thereof were recorded separately. In total, 4 days and 7 hours of 16-channel audio data has been recorded with the head and torso simulator.

The head circumference and width as well as the pinna length and pinna width of the head and torso simulator can be found in the “Demography.csv”.

## Technical Validation

During the data processing and development of the acoustic Cocktail Party dataset, verification and validations were made at several stages: As a first step, each of the 12 loudspeakers was calibrated with a free field microphone (NTi, Audio M2211, Schaan, Liechtenstein) positioned in the center of the circular setup and an audio analyzer (NTi, Audio XL2, Schaan, Liechtenstein). Afterwards, to ensure a wide range of subjective SNR values, hearing tests of the audio source files and the recordings of pilot measurements were conducted. During the file generation process, all audio files were checked for clipped samples. Only audio files with less than 1% of clipped samples were included in the dataset. After the file generation process, overview tables as described in Table 10 and the *Data Records* section, were created. A detailed analysis of the created CPCs and their components with regard to their validity was performed (see Tables 5 and 8). Since the attached .csv files provide full transparency regarding the SNR values of the generated files and machine learning algorithms may benefit from a slight variance of the data^57^, no adjustment was made to the generated data.

**Table 8:** The data shows an overview of the SNR values and one-standard deviations for the multichannel source files in the head and torso simulator measurements.

Noise	SNR (Loudness setting ID = 0)			SNR (ID = 1)				SNR (ID = 2)	
Filename prefix	1 speaker	2 speakers	3 speakers	2 speakers		3 speakers		3 speakers	
				loud target	silent target	loud target	silent target	loud target	silent target
CS1	no noise	0.0 + −0.1	−2.7 + −0.2	15.0 + −0.0	−14.9 + −0.1	−0.1 + −0.2	−17.6 + −0.2	12.1 + −0.3	−15.0 + −0.2
MU1	15.1 + −0.1	0.0 + −0.1	−2.8 + −0.2	15.1 + −0.2	−14.9 + −0.1	0.0 + −0.1	−17.6 + −0.2	12.2 + −0.1	−14.9 + −0.3
MU2	15.1 + −0.1	0.0 + −0.1	−2.8 + −0.2	14.6 + −0.3	−14.9 + −0.0	−0.1 + −0.1	−17.7 + −0.1	12.1 + −0.1	−14.9 + −0.1
MU3	15.1 + −0.1	0.0 + −0.1	−2.8 + −0.2	14.8 + −0.1	−14.8 + −0.1	0.0 + −0.2	−17.5 + −0.2	12.3 + −0.4	−14.9 + −0.2
MU4	15.1 + −0.1	0.0 + −0.1	−2.8 + −0.2	14.8 + −0.0	−14.9 + −0.1	−0.1 + −0.2	−17.6 + −0.1	12.2 + −0.1	−15.0 + −0.1
MU5	12.1 + −0.1	−0.1 + −0.2	−2.8 + −0.2	14.8 + −0.2	−14.9 + −0.1	0.0 + −0.1	−17.7 + −0.1	12.0 + −0.2	−14.9 + −0.2
MU6	12.1 + −0.1	−0.1 + −0.2	−2.8 + −0.2	14.9 + −0.2	−14.9 + −0.1	−0.2 + −0.1	−17.5 + −0.2	12.1 + −0.3	−14.9 + −0.2
MU7	9.1 + −0.1	−0.2 + −0.2	−2.9 + −0.2	14.6 + −0.1	−15.0 + −0.0	0.0 + −0.2	−17.7 + −0.2	12.0 + −0.2	−15.0 + −0.1
BA1	20.5 + −0.1	0.0 + −0.1	−2.8 + −0.2	14.9 + −0.1	−14.9 + −0.1	0.0 + −0.2	−17.6 + −0.3	12.2 + −0.2	−15.0 + −0.1
BA2	20.5 + −0.1	0.0 + −0.1	−2.7 + −0.2	15.0 + −0.0	−15.0 + −0.0	0.0 + −0.2	−17.6 + −0.3	12.1 + −0.1	−15.0 + −0.1
BA3	20.5 + −0.1	0.0 + −0.1	−2.7 + −0.2	15.0 + −0.1	−14.9 + −0.0	−0.1 + −0.1	−17.7 + −0.2	12.2 + −0.2	−14.9 + −0.2
BA4	20.5 + −0.1	0.0 + −0.1	−2.7 + −0.2	15.0 + −0.1	−14.9 + −0.1	0.0 + −0.2	−17.7 + −0.2	12.1 + −0.2	−15.0 + −0.1
BA5	14.5 + −0.1	−0.1 + −0.1	−2.8 + −0.2	14.9 + −0.0	−14.9 + −0.1	−0.2 + −0.2	−17.6 + −0.2	12.2 + −0.2	−15.0 + −0.0
MB1	11.8 + −0.1	−0.1 + −0.3	−2.8 + −0.2	14.8 + −0.1	−15.0 + −0.0	−0.1 + −0.1	−17.6 + −0.1	12.1 + −0.3	−15.0 + −0.1
MB2	11.8 + −0.1	−0.1 + −0.1	−2.8 + −0.2	14.7 + −0.7	−14.7 + −0.4	−0.1 + −0.2	−17.5 + −0.2	12.2 + −0.2	−15.0 + −0.1
MB3	11.8 + −0.1	−0.1 + −0.1	−2.8 + −0.2	14.8 + −0.1	−14.9 + −0.1	0.0 + −0.2	−17.6 + −0.2	12.0 + −0.2	−15.0 + −0.2
MB4	11.8 + −0.1	−0.1 + −0.2	−2.8 + −0.2	14.8 + −0.1	−14.9 + −0.1	−0.1 + −0.2	−17.5 + −0.3	12.1 + −0.2	−14.9 + −0.2
MB5	10.1 + −0.1	−0.1 + −0.3	−2.9 + −0.2	14.8 + −0.2	−14.7 + −0.4	−0.1 + −0.2	−17.7 + −0.2	12.0 + −0.2	−14.9 + −0.3
MB6	10.1 + −0.1	−0.1 + −0.3	−2.9 + −0.2	14.6 + −0.2	−14.8 + −0.1	−0.1 + −0.2	−17.6 + −0.2	12.0 + −0.1	−14.8 + −0.4
MB7	8.0 + −0.2	−0.3 + −0.2	−3.0 + −0.2	14.6 + −0.2	−14.9 + −0.1	−0.1 + −0.2	−17.6 + −0.2	11.9 + −0.1	−15.0 + −0.2

The noise prefixes are defined in Table 4. A description of the loudness setting ID can be found in Table 9.

To avoid the influence of unwanted movements during the sound presentation, the participants were instructed to fixate a frontally mounted monitor (Surface Book, Microsoft, USA) on which a silent movie of their choice was shown. The position of the monitor showing the silent movie ensured that the head position of the participant was maintained as required. To further mitigate the impact of undesired movements during data collection, the test persons were monitored by a camera (USBFHD06H-BFV, ELP, China), which was mounted on the ceiling of the acoustic chamber. In case of movements of the participants, the recording was prematurely interrupted by the investigator. After each recording, a random selection of the 16-channel audio files generated during a successful recording session were systematically checked for possible technical signal interference using channel-by-channel listening tests.

To ensure the validity of the acquired 3D coordinates of the microphone positions, the Euclidean distances between all microphones were calculated for each participant and stored in a microphone-distance matrix. Afterwards the matrices were compared to each other and outliers were examined. Outliers may occur, for example, due to inaccuracies in the manual annotation of the microphone positions. As cut-off we chose three standard deviations from the mean as it is a common practice for identifying outliers in a Gaussian or Gaussian-like distribution. Apart from outliers in between the participants, the plausibility of microphone spacing was also checked individually for each participant with a Python script.

Our measurements of the external ear and the head sizes were compared with results from the literature^58,59^. The compared to Bozkir *et al*.^58^ 4 mm decreased average ear length may be due to the slight curvature of the pinna caused by the CI audio processor.

## Usage Notes

The audio files of the CPCs are divided into recordings with human participants and recordings with the head and torso simulator. At the root folder level of the dataset for each human participant audio recording, the collection of CPCs is divided into the same 12 sub-scenarios. Each of these 12 scenarios is characterized by the number of dominant speech sources (1, 2 or 3) and the type of background noise (music, babble noise, music and babble noise or silence), resulting in 3 × 4 = 12 sub-scenarios. For the recordings with the head and torso simulator, each of the 12 sub-scenarios is provided as a separate archive.

The folders inside each sub-scenario follow a specific name structure that defines the detailed Cocktail Party scenario to which its file content refers. The naming of these sub-folders follows the structure AAB_CDDDEEEF_GG and is explained in Table 9.

**Table 9:** Explanation to the folder names (AAB_CDDDEEEF_GG) that define an acoustic Cocktail Party scenario setting.

Identifier	Description	Values
AA	Noise ID	CS = Clean Speech, MU = Music, BA = Babble, MB = Music and Babble noise.
B	Noise setting number	Numeric. Serves to group the noise composition e.g. the spatial arrangement of the music sources.*
C	Number of speech sources	Numeric (1 to 3).
DDD	Azimuth offset between the speech sources	Numeric (030, 060, 090 or 120 degree angle in clockwise direction).
EEE	Reference azimuth for the speech sources	Numeric. Starting azimuth for the clockwise shift of the speech sources.
F	Loudness setting ID	Numeric (0 to 2). Serves to group the different loudness-level settings of the speech sources.
		0: All speech sources are equally loud; 1: Alternating level in LUFS of the speech sources: −15, -30, −15, …; 2: Alternating level in LUFS of the speech sources: −30, −15, −30, …
GG	Sample number	Numeric. Indicates the sample number of a Cocktail Party scenario as defined by A-F.

Folders with names of this structure contain all audio files of a Cocktail Party setting.

*Definitions on the noise setting number can be found in Tables 4 or 7 for the head and torso simulator.

The CPC folders with the structure as explained in the Table 9 contain all audio and text descriptions of a CPC as described in the *Methods* section. The 12-channel audio source files for the compiled CPC can be found inside the folder “mixture” and the audio components of this mixture are in the folder with the name “components_of_the_mixture”. Within the components of the mixture files, “_B” indicates babble noise, “_M” music and “_S*N*” clean speech of the speaker on channel *N*. Combinations of components are self-explanatory based on the file names. Within the components and the mixture folders, the 16-channel recording files are inside the “Recordings” folder and their filename starts with “rec_“. All recordings are stored with a sample rate of 44.1 kHz and an audio bit depth of 32 bit.

In addition to the folders with the CPCs, which contain the audio files and text descriptors, overview tables for the CPCs are provided in the form of .csv files. The tables describe the acoustic composition of each CPC at channel level. The meaning of the column names is explained in the Table 10.

**Table 10:** Description of the metrics in the.csv-tables which describe the recorded Cocktail Party settings.

Column name	Description
Noise_Id	see AA in Table 9
Noise_setting_num	see B in Table 9
Num_of_speech_sources	see C in Table 9
Deg_between_ss	see DDD in Table 9
Position_ref_ss	see EEE in Table 9
dB_mod_Id	see F in Table 9
Sample	see GG in Table 9
Channel	Channel number
Music_file	Unique ID of the music file taken from the Musan corpus^41^. Attached to the file name is the time window of the file section in ms.
Music_dBLUFS_orig	Level in LUFS of the music file.
Music_dBLUFS_out	Level in LUFS of the music file as applied to the mixture.
Babble_file	Unique ID of the babble noise file from our generated babble noise corpus.
Babble_dBLUFS_orig	Level in LUFS of the babble file.
Babble_dBLUFS_out	Level in LUFS of the babble file as applied to the mixture.
Speech_file	Unique ID of the speech file taken from the LibriTTS corpus^40^.
Speech_dBLUFS_orig	Level in LUFS of the speech file.
Speech_dBLUFS_out	Level in LUFS of the speech file as applied to the mixture.
SNR_single_channel	Ratio between the level in LUFS of the speech file and the level of music and babble noises on this channel.
SNR_all	Ratio between the level in LUFS of the speech file on this channel and the level of all remaining sounds.
SNR_all_weighted	See *SNR_all*. Weighting refers to the direction dependent channel gains as specified in ITU-R BS.2051^56^.
dBLUFS_single_channel	Channel level in LUFS.
dBLUFS_single_channel_weighted	Weighted channel level in LUFS as specified in ITU-R BS.2051^56^.
dBLUFS_all	Level of the mixture in LUFS.
dBLUFS_all_weighted	Weighted level of the mixture in LUFS as specified in ITU-R BS.2051^56^.
Filename	Unique ID of the created Cocktail Party scenario mixture.

For using the code as described in the section *Code availability*, we provided an .yml file alongside with the code to install all dependencies necessary to run the provided python code. The files in the Hardware folder can be viewed with the free version of EAGLE or, to reproduce the hardware, sent to a PCB assembly service. The individual microphone positions and distances for each measured participant are provided as .csv files.

Many algorithms for speech signal enhancement use methodologies that are designed to work with a predefined distance matrix of microphones^60^. The use of an “average” participant can be useful for the time being, since an averaged value of the microphone distances certainly represents a plausible starting point. Nevertheless, for a validation of the stability of the developed methodologies we would recommend to consider the performance based on the results of individual participants.

## Data Availability

The code used to create and process the presented data is provided in^55^ or is part of open source repositories.
